# The Effect of Heterogeneous Definitions of Massive Transfusion on Using Blood Component Thresholds to Predict Futility in Severely Bleeding Trauma Patients

**DOI:** 10.3390/jcm14155426

**Published:** 2025-08-01

**Authors:** Samuel J. Thomas, Vraj S. Patel, Connor P. Schmitt, Aleksey T. Zielinski, Mia N. Aboukhaled, Christopher A. Steinberg, Ernest E. Moore, Hunter B. Moore, Scott G. Thomas, Dan A. Waxman, Joseph B. Miller, Connor M. Bunch, Michael W. Aboukhaled, Emmanuel J. Thomas, Saniya K. Zackariya, Halina Oryakhail, Alexander Mehreteab, Reagan E. Ludwig, Sarah M. George, Aayan I. Siddiqi, Bilal M. Zackariya, Aadil Qasim, Mark M. Walsh, Mahmoud D. Al-Fadhl

**Affiliations:** 1 Department of Emergency Medicine, Saint Joseph Regional Medical Center, Mishawaka, IN 46545, USA; 2 Ernest E. Moore Shock Trauma Center, Denver Health, Denver, CO 80204, USA; 3 Department of Surgery, University of Colorado Health Science Center, Aurora, CO 80045, USA; 4 Beacon Medical Group Trauma & Surgical Research Services, South Bend, IN 46601, USA; 5 Versiti Blood Center of Michigan, Grand Rapids, MI 49503, USA; 6 Department of Pathology and Laboratory Medicine, Indiana University School of Medicine, Indianapolis, IN 46202, USA; 7 Department of Emergency Medicine, Henry Ford Hospital, Detroit, MI 48202, USA; 8 Indiana University School of Medicine, Northwest-Gary Campus, Gary, IN 46408, USA; 9 Indiana University School of Medicine, South Bend Campus, South Bend, IN 46617, USA

**Keywords:** medical futility, futile resuscitation, massive transfusion, blood component transfusion, trauma, hemorrhage, algorithm, red blood cells

## Abstract

In the trauma resuscitation literature, there are inconsistent definitions of what constitutes massive transfusion and a unit of blood, complicating the use of transfusion cut-points to declare futility. This is problematic as it can lead to the inefficient use of blood products, further exacerbating current blood product shortages. Previous studies have used various transfusion cut-points per hour to define futility in retrospective analyses but have not accurately defined futility at the bedside due to patient survival even at large rates and volumes of blood transfused. In an attempt to use transfusion cut-points as a marker to help define futility, guidelines have been proposed to limit blood product waste in transfusions for severely bleeding trauma patients, such as Suspension of Transfusion and Other Procedures (STOP) for patients older than 15 and the Futility of Resuscitation Measure (FoRM), used to determine futility in patients older than 60. In an effort to construct effective bedside futile resuscitation criteria with 100% positive predictive value and specificity, this review proposes the use of specific blood component transfusion cut-points combined with parameters from both STOP and FoRM to allow for a comprehensive and accurate method of declaring futility in severely bleeding trauma patients.

## 1. Introduction

Currently, in the United States and worldwide, there has been a shortage of blood products available at major blood banking institutions. The unexpected disruption of the world’s blood supply caused by the COVID-19 pandemic created a vacuum of donors and localities for the donation, collection, and processing of blood products due to the disruption in the blood banking process, largely in part due to impacted at-work blood drive programs [1,2,3,4,5,6,7,8,9]. Not only did this reduce the opportunity for blood bank-sponsored collection, but it also reduced the number of donors, which had been dwindling even before the pandemic [10]. Coincident with this COVID-19-associated phenomenon, increased use of balanced hemostatic resuscitation in the form of 1:1:1 packed red blood cells (PRBCs)–fresh frozen plasma (FFP)–platelets (PLTs) with an increased adoption of whole blood (WB) and other adjunctive blood component therapies further limited the amount of blood products available during times of peak use [11,12,13].

Because of these phenomena, there have been numerous attempts to quantitatively define futile resuscitation (FR) during the early stages of the administration of blood products and accurately predict death in severely bleeding trauma patients (SBTPs) [4,6,11,12,13,14]. Specifically, Suspension of Transfusion and Other Procedures (STOP) has been proposed as a reliable predictor of death with 100% positive predictive value (PPV) and specificity [13]. A similar type of algorithm for quantitatively predicting FR in SBTPs called the Futility of Resuscitation Measure (FoRM) assigns a point value for the likelihood of futility based on the presence of traumatic brain injury (TBI), age over 60 stratified by decade, and other markers of the depth and duration of shock [15]. The importance of these attempts to quantify futility during the use of massive transfusion (MT) for SBTPs is that they can be used at the bedside and could alleviate part of the source of blood shortages, which have followed the use of balanced hemostatic resuscitation and WB in the early moments of resuscitation.

This attempt to quantify futility has led to studies using blood product cut-points per hour as reliable predictors of death [11,12,16,17,18,19,20,21,22,23]. However, there has been significant controversy about whether a threshold exists [11,12,22,24]. Complicating the attempts to use transfusion cut-points as reliable markers of futility is the fact that different institutions use different definitions of a unit of blood product and MT; while some institutions only count the amount of PRBCs transfused per hour, others may include FFP, PLTs, WB, as well as total volume of blood transfused per varying amounts of time [3,11,12,17,22,25,26,27,28,29,30,31]. A universal definition of MT would help in the early identification of which patients will require MT, allowing traumatologists from various settings to refine their anticipation of futility [32]. The confusion wrought by varying definitions of MT can be demonstrated by a description of the evolution of these protocols into traditional and modern versions [27,28].

Identifying markers for stopping MT during FR is critical, since stopping transfusion prematurely can lead to the death of the patient, and stopping transfusion late can lead to wasting blood products when there is no chance of survival. These markers should aim to have close to 100% PPV and specificity, ensuring almost no false positives in predicting death from traumatic injury. Although defining parameters cannot totally replace clinical gestalt, this paper provides a review of previously proposed and potentially more precise markers to aid the clinician in ceasing resuscitation while also giving reason to the lack of uniformity in the literature for the definition of MT and what constitutes a unit of blood component [11,12,13,14,33,34].

The purpose of this review is to trace the historical evolution of the definitions of MT related only to trauma and to propose a more homogeneous definition, which would be of use not only at the bedside but also for larger randomized controlled trials (RCTs) that would use the markers of MT to compare morbidity and mortality of various trauma protocols [35]. Additionally, a protocol to address the declaration of futility for SBTPs, which combines the use of STOP and FoRM with reference to other protocols that highlight the importance of age and presence of TBI, is proposed.

## 2. Blood Component Cut-Points of Transfusions to Define Futility

### 2.1. Historical Evolution of Defining MT Based on Units of Blood Component per Hour

The markers that allow a traumatologist to predict FR are often the same as those used to guide the initiation and definition of MT, which historically was defined as providing a replacement of an adult’s total blood volume, which is ≥10 units (U) PRBCs within 24 h, and RCTs have solidified this definition in the trauma literature [35,36]. Additionally, recent definitions of MT have been more varied in both units of blood components transfused and units of time to receive those blood components [27,28]. Terms such as ultramassive transfusion (UMT), typically reported as ≥20 U PRBCs in 24 h, have been based on the traditional definition of MT, yet more recently, UMT has also undergone a similar evolution in terms of the type and units of blood components over varying times from 1 to 24 h [11,18,25,28,29].

### 2.2. Critical Administration Threshold (CAT) and Resuscitation Intensity (RI)

Another measure for blood transfusion is CAT, which describes the number of times within a 24 h period that the rate of transfusion exceeds 3 U PRBCs/hour. For example, a patient who receives ≥3 U of PRBCs in four distinct one-hour periods during the first 24 h of resuscitation would be considered CAT-4. A higher numerical suffix identifies patients who will need more PRBCs in the face of uncontrolled hemorrhage [37,38,39,40].

Additionally, to further refine the definition and triggers for MT, a prediction tool called RI has been proposed to define the amounts of PRBCs, FFP, PLTs, crystalloid (with 1000 mL equivalent to 1 U blood component), and colloid (with 500 mL equivalent to 1 U blood component) given in a 30 min timespan. The number of units transfused in those 30 min are appended as a suffix. For example, RI4+ is identified as the use of ≥4 U blood components, crystalloid, and colloid in 30 min [31,37,38]. Despite demonstrating particular sensitivity as a predictor of death in patients in hemorrhagic shock, the RI score is difficult to compare to other transfusion definitions and predictions for the need of MT, since all blood products are considered, which contrasts with previous studies using only PRBCs per hour [31,37,38,41]. The CAT and RI scores are examples of prediction tools that are useful for retrospective analyses of varying resuscitation strategies but can be more cumbersome at the bedside during resuscitation because they depend on discrepant definitions of the interval of transfusion or blood components considered. The CAT score has been advocated as a superior definition of MT for purposes of predicting mortality when compared to the use of PRBCs in 24 h. However, the use of PRBCs in this study was the older figure of 10 U/24 h, which has been known for years to not effectively predict mortality [42].

The transfusion rate of PRBCs per hour has been the most commonly studied marker of FR, with varying sentiments on its effectiveness [43,44,45,46,47,48]. A major problem for establishing standardized blood transfusion per hour cut-points of FR is that the literature defines the composition of a unit of blood in various ways, which have evolved over time like the definition of MT has evolved [42,49,50]. Due to the non-standardized methods of defining those who need MT and those who will have FR, none of these definitions based on PRBCs or blood components have allowed for an accurate anticipation of the patients who would certainly die. The search for better markers to define MT has led to an analysis of the amounts of blood components transfused in certain amounts of time such as the CAT and RI algorithms. While the RI algorithm accounts for the quantity of crystalloid and colloid in its calculation, neither RI nor CAT addresses the recent rise in the use of WB in urban American trauma centers [11,37,50,51,52,53,54,55,56,57,58,59,60,61,62,63]. The result of the prehospital and hospital trauma protocols that now use WB complicates the creation of a transfusion cut-point marker, which would define patients in need of MT and identify SBTPs for whom resuscitation would be futile. The incorporation of WB in algorithms of predicting MT and futility has led to a score that requires the tallying of WB with PRBCs to predict early mortality at the bedside [37].

### 2.3. Incorporation of WB as Part of a Cut-Point

For those centers that use WB, a separate scoring system, which provides extra weight for WB when compared to PRBCs, has been defined as the WB MT score where WB MT = 3 × U WB + U RBC [37]. The weighted inclusion of WB for MT has confounded comparisons of WB resuscitation with 1:1:1 PRBCs–FFP–PLTs transfusion practices, as well as the literature for predicting early mortality. For early identification of futility, a proposal would be to simply count 1 U WB as 1 U PRBC for the purposes of calculating a futility index at the bedside. A rationale for this strategy is that 1 U WB and 1 U PRBCs have approximately similar volume and storage, and for those patients who receive MT, 1 U PRBCs is associated with 1 U PLTs and 1 U FFP, which approximates the physiological function of 1 U WB [64].

### 2.4. Simplification of Defining Blood Components for FR

MT and UMT have been commonly defined in PRBC U/hour. However, in many studies, WB was also included in the definition [48,65,66]. Other studies use the term “blood” but do not specify whether the transfused components were PRBCs alone or in combination with FFP and PLTs [3,67,68,69]. Additionally, the term total transfusion volume has been used recently [20]. There is no universal cut-off for futile transfusion, either in the type of blood component or per unit of time [3,11,18,22,23]. As a result, there has been a significant discrepancy not only in defining UMT but also in defining cut-points, which indicate FR in the patient with traumatic hemorrhagic shock. To simplify and create a more operational definition of MT, a European group conducted a Delphi analysis to reach a consensus definition of major bleeding in trauma by establishing new criteria that incorporate volume and rate of transfusion in a smaller time window, which reduces bias. This European consensus felt that a definition of MT should include all types of blood components and not just PRBCs. This European group also agreed that the transfusion of ≥4 U of any blood component including WB within 2 h of injury should define MT and is consistent with the evolution of transfusion practices for traumatic hemorrhage, which emphasizes the immediate administration of all types of blood components [18,41,70]. The heterogeneous and evolving definitions of MT solely in trauma are summarized in Table 1.

Recent review of traditional RCT definitions of MT based on PRBCs alone have identified heterogeneity in the number of PRBCs/hour with a majority of the papers relying on the older and less sensitive definition of 10 U PRBCs in 24 h [35]. Earlier literature on the correlation between the number of units of blood products given per hour and mortality has mostly relied on PRBCs [41,44,46,70]. While there remains some adherence to using only PRBCs to define MT, the trend in some of the recent literature is to include all blood components per hour with or without WB [3,11,13,22,50,70,75]. This ambivalence in the literature regarding the definition of MT and UMT is reflected in the most recent attempts to utilize transfusion cut-points as predictors of FR, and has been amplified by the suggestion that MT be separated into traditional MT and modern MT [27,28].

Because of the many inconsistencies demonstrated in Table 1, it would seem logical to utilize a straightforward method of defining MT for the identification of transfusion cut-points as predictors of FR with the easiest and most reproducible parameters of blood component administration during trauma resuscitation. A summary of the salient papers that have defined these cut-points would be beneficial in helping to define a more homogeneous definition of FR in SBTPs.

### 2.5. Summary of Literature Defining Transfusion Cut-Points as Predictors of FR

Table 2 summarizes the literature of these blood component cut-points per hour in trauma. They are mostly in PRBCs per hour, but recently, cut-points using total blood components per hour (including WB) have become more commonly used. These are the foundations upon which other markers are applied in various studies and algorithms to predict FR.

After trauma, rapid and disorganized administration of varying blood components within a short time frame presents challenges in accurately tracking transfusions. Electronic counters or stacking empty unit bags have been used as systems to record real-time blood transfusion at the time of MT [99].

Using cut-points for blood product administration is a straightforward method when using a whiteboard, which can be transported with the patient to keep track of blood products consumed. During transfusion, the accuracy of predicting FR can be improved through the use of immediately available bedside markers. The major issue with these clinical parameters is that some, such as the Glasgow Coma Scale (GCS), can be affected by paralysis and sedation in intubated patients. Others cannot be calculated immediately, such as the sequential organ failure assessment (SOFA), injury severity score (ISS), and abbreviated injury severity score (AIS) [4,100,101]. Additionally, obtaining and processing metabolic and coagulation data requires time, making them impractical for bedside use during resuscitation [101,102].

## 3. Combination of Clinical, Laboratory, and Transfusion Cut-Point Markers to Determine Futility

**Figure 1:**
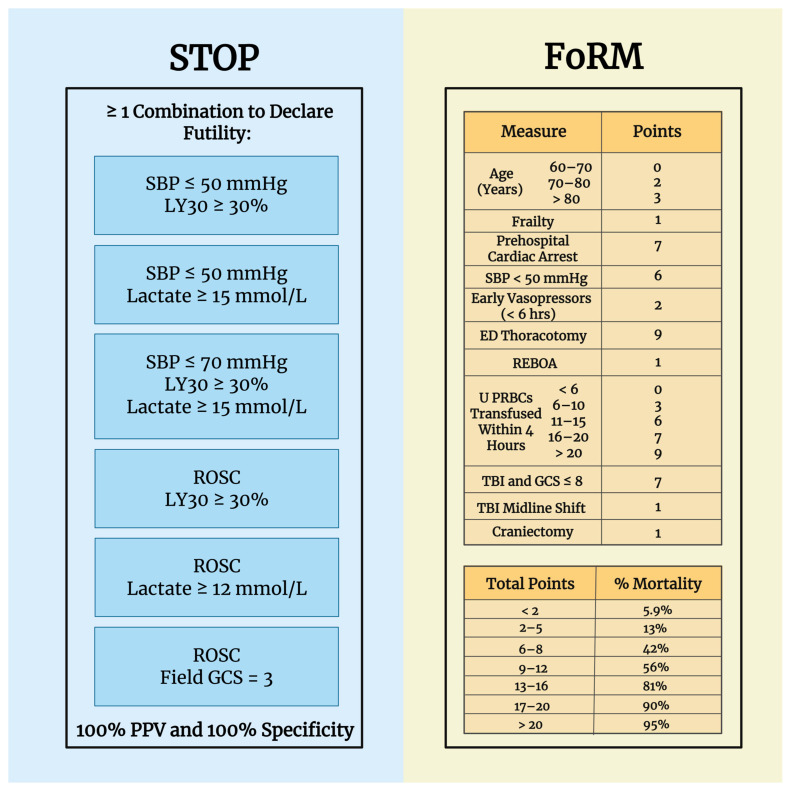
A summary of the STOP and FoRM criteria [13,15]. Abbreviations: ED (emergency department), FoRM (Futility of Resuscitation Measure), GCS (Glasgow Coma Scale), LY30 (lysis at 30 min), PRBC (packed red blood cell), REBOA (resuscitative endovascular balloon occlusion of the aorta), ROSC (return of spontaneous circulation), SBP (systolic blood pressure), STOP (Suspension of Transfusion and Other Procedures), TBI (traumatic brain injury). Created in BioRender. Al-fadhl, M. (2025) https://BioRender.com/bo67sz0 (accessed on 18 June 2025).

The search for bedside algorithms for declaring FR has been emphasized as a means of reducing the amount of blood products wasted during periods of blood shortage in blood banks across the nation. Recently, the STOP criteria have been established as a method of declaring FR with 100% PPV and specificity [13]. FoRM is another method that could be implemented as a bedside algorithm to declare futility in SBTPs who are ≥60 years. In both derivation and validation cohorts, FoRM exhibited high area under the receiver operating characteristic (AUROC) values (0.860 and 0.76, respectively), demonstrating its reliability in predicting FR with patients scoring above 20 having less than 5% chance of survival [15]. As summarized in Figure 1, STOP relies on parameters such as lactate, systolic blood pressure (SBP), return of spontaneous circulation (ROSC), GCS, and LY30, and FoRM utilizes patient information including age, frailty, cardiac arrest, ≥1 episode of low SBP, use of vasopressors within 6 h, thoracotomy, resuscitative endovascular balloon occlusion of the aorta, U PRBCs within 4 h, severe TBI and GCS ≤ 8, and craniectomy. Additionally, STOP can be applied to all adults, but FoRM is reserved for adults 60 or older [13,15].

## 4. Proposal of Protocol for Guiding Declaration of FR for SBTPs

There has been controversy regarding the utilization of blood products per hour as cut-points to reliably predict death for SBTPs who did not respond to resuscitation [11,12]. However, these commentaries have not included subset analyses of patients with cranial and extracranial injuries stratified by age. Because of the increased number of elderly patients in motor vehicle collisions who present to the trauma center with cranial and extracranial injury, the use of blood products per hour in the first hours of MT in this group of patients with severe TBI would serve as a reliable marker to predict futility. Therefore, a coherent and uniform method of documenting the number of blood products used in these situations would facilitate a standard protocol for withdrawing care for this specific subset of patients [15,103]. In addition, the use of a standardized method for defining MT would assist in studies comparing different institutions for all SBTPs. Finally, exclusively counting PRBCs when providing MT is easier than counting every blood component transfused. While the STOP criteria do not include PRBC cut-points, the FoRM criteria assign higher point values to greater volumes of transfusion within 4 h [13,15]. Even though cut-points alone may not be enough to make a decision regarding FR, their incorporation into a scoring system with other markers such as TBI has shown efficacy [15].

**Figure 2:**
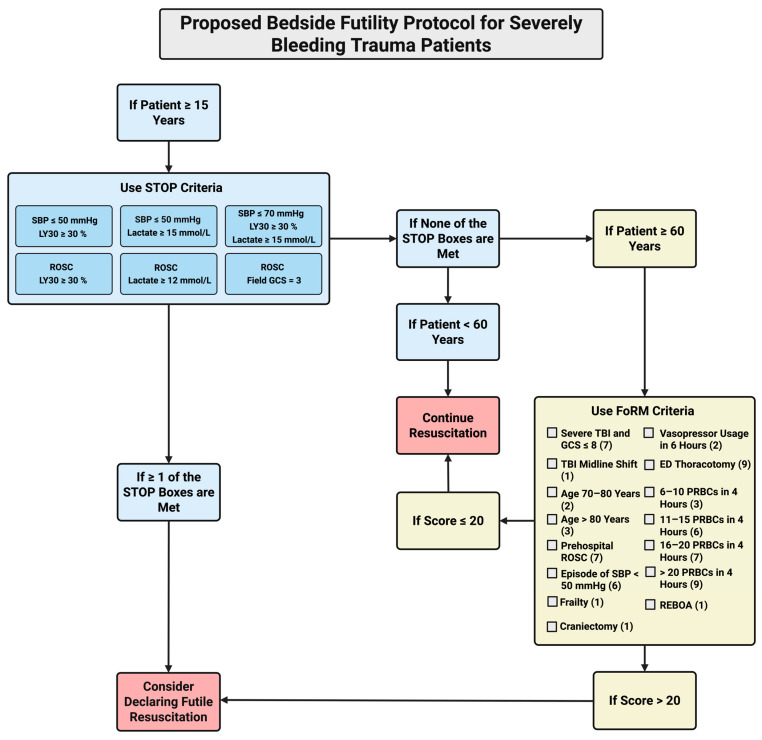
A flowchart of suggested steps, based on STOP and FoRM protocols, when faced with the decision to declare futility in bleeding trauma patients. Abbreviations: FoRM (Futility of Resuscitation Measure), GCS (Glasgow Coma Scale), LY30 (lysis at 30 min), PRBC (packed red blood cell), REBOA (resuscitative endovascular balloon occlusion of the aorta), ROSC (return of spontaneous circulation), SBP (systolic blood pressure), STOP (Suspension of Transfusion and Other Procedures), TBI (Traumatic Brain Injury) Created in BioRender. Al-fadhl, M. (2025) https://BioRender.com/n6lg8hm (accessed on 18 June 2025).

We propose a theoretical method for declaring FR for SBTPs that incorporates both STOP and FoRM criteria. Integrating FoRM criteria into a protocol that uses STOP includes TBI severity and transfusion cut-points as additional markers that may improve the accuracy of declaring FR (Figure 2). The initial step for adult SBTPs in the flowchart is to use the STOP criteria, as they are inclusive of all SBTPs above the age of 15, have 100% PPV, and have 100% specificity in predicting futility [13]. However, they will likely only pertain to select SBTP cases. If no STOP criteria are met, FoRM can be applied to patients 60 or older. If the FoRM score is greater than 20, declaring FR should be considered. This flowchart is an example of a process that could facilitate the decision to declare futility at the bedside. Further studies would be necessary to validate the efficacy of similar flowcharts in the declaration of FR in the trauma setting.

## 5. Conclusions

Definitions of MT are conflicting, which leads to confusion about management of blood products when treating SBTPs. This is further complicated by what is defined as a transfused unit of blood product. In this review, we discuss how these inconsistencies lead to ineffective determination of transfusion cut-points for identifying when to declare FR. This prompts the establishment of reliable guidelines for the determination of futility that do not solely rely on the number of blood products per hour. By combining STOP and FoRM criteria, we propose one possible protocol to establish whether FR should be declared or resuscitation efforts should be continued.

## Figures and Tables

**Table 1:** Definitions of massive transfusion and other transfusion thresholds in trauma.

Name	Quantity of Blood Given
Massive Transfusion (MT)	≥3 U packed red blood cells (PRBCs) in 1 h [71,72]
	>4 U PRBCs in 1 h [73]
	≥4 U PRBCs in 1 h [27,74,75]
	≥4 U blood components in 2 h [70]
	≥4 U PRBCs in 6 h [76]
	>5 U blood products in 4 h [24]
	>6 U PRBCs in 2 h [73]
	≥4 U of PRBCs, ≥1 U of plasma, and ≥1 U of platelets (PLTs) within 4 h [17]
	>6 U PRBCs in 48 h [77]
	>8 U PRBCs in 4 h [73]
	≥10 U PRBCs in 4 h [12]
	≥10 U PRBCs in 6 h [27,78]
	>10 U PRBCs in 6 h [73]
	≥10 U PRBCs in 24 h [3,21,24,45,49,71,72,74,75,79,80,81,82,83,84,85,86,87,88]
	>10 U PRBCs in 24 h [27,89]
	>10 U PRBCs [90]
	>12 U PRBCs in 12 h [73]
	>12 U PRBCs in 24 h [73]
	>20 U PRBCs [36,91]
	Replacement of half of patient’s entire blood volume within 3 h [21,36]
	Replacement of patient’s entire blood volume within 24 h [36]
	Transfusion at rate of >150 mL/min [21]
Ultramassive Transfusion (UMT)	≥20 U PRBCs in 4 h [3,12,18,19]
	≥20 U blood components in 24 h [3,22,25,26,29,30]
Dynamic MT	Transfusion of ≥4 U PRBCs in 1 h when ongoing need is foreseeable [36]
Supermassive Transfusion (SMT)	≥25 U PRBCs in 24 h [92]
	≥50 U blood components [22]
Critical Administration Threshold (CAT)	CAT+ if ≥3 U PRBCs within a single hour [39,40,93,94]
	CAT-X where X is either 1 h, 4 h, or 24 h and represents the time to transfuse ≥3 U PRBCs [27]
	CAT-X where X is the number of times a patient exceeds CAT (≥3 U PRBCs within a single hour) in 24 h [39,40]
Resuscitation Intensity (RI)	RI+ if ≥4 U PRBCs, fresh frozen plasma (FFP), PLTs, crystalloid (1000 mL equivalent to 1 U), and colloid (500 mL equivalent to 1 U) within 30 min [31]
	RIX where X is the number of units and 1 U = 1 L crystalloid solution, 0.5 L colloid, 1 U PRBCs, 1 U plasma, or 6 U PLTs transfused in 30 min [31]
Whole Blood Massive Transfusion (WB MT) Score	(3 × U WB) + U RBC within the first hour [37]
	WB MT (+) = WB MT score ≥ 7 WB MT (−) = WB MT score < 7 [37]

**Table 2:** Summary of studies on transfusion cut-points as predictors of futile resuscitation in trauma.

Study	Design and Population	Results and Conclusions
Siegel et al. (1990) [95]	Retrospective study of 185 patients with major hepatic injury caused by blunt trauma	The authors found that base excess (median lethal dose [LD50] = −11.8 mmol/L) and blood volume transfused within the first 24 h (LD50 = 5.4 L) were significant predictors of death. A predictive model they generated using Glasgow Coma Scale and base excess was highly successful in predicting death.
Cosgriff et al. (1997) [96]	Retrospective observational study of 58 patients in a two-year period who were older than 15 years (mean = 35.4 years), did not have pre-existing disease or massive head injuries, and received massive transfusion (MT) (>10 U packed red blood cells (PRBCs) in 24 h).	The authors found that U PRBCs/6 h and U PRBCs, U fresh frozen plasma (FFP), and U platelets (PLTs)/24 h were not significant predictors of mortality.
Velmahos et al. (1998) [48]	Retrospective observational study of 141 trauma patients (mean injury severity score [ISS] 29, penetrating injury 74%, mortality rate 30.5%) receiving >20 U of PRBCs in the preoperative and intraoperative period. One unit was defined as 1 U of WB or 1 U PRBCs.	The number of units transfused did not differ between survivors and non-survivors. Volume of blood loss and transfusion was less critical than quality and duration of shock. No single clinical, laboratory, or procedural marker was predictive of futility. However, out of a cohort of 13 patients, aortic clamping, use of inotropes, and 90+ minutes of hypotension combined resulted in 100% mortality. The authors concluded that discontinuation of MT cannot be justified for up to 68 U blood.
Vaslef et al. (2002) [69]	Retrospective observational study of 44 patients (mean ISS 36.8, blunt injury 61.4%) who received >50 U of all blood components in the first 24 h of admission.	Patients receiving >50 U of blood products had a survival rate of 43%, and thus aggressive transfusion should continue for those trauma patients requiring >50 U at 24 h after admission. The authors concluded that volume and/or total units of blood products in the first 24 h were not independent risk factors for mortality.
Rangarajan et al. (2011) [97]	Retrospective observational study analyzing 71 trauma patients (median ISS = 27) who received MT, defined as ≥10 U of PRBCs in 24 h, in a Level I trauma center	The authors concluded that the PRBC units transfused in the first 12 h as well as the total number of blood products transfused were not significant indicators of in-hospital mortality.
Liu et al. (2018) [45]	Retrospective observational study analyzing 131 adult trauma patients who received blood transfusion. Mortality was 24% for patients who received 0–9 U PRBCs, 21% for 10–19 U, 38% for 20–29 U, 50% for 30–39 U, and 80% for ≥40 U.	The authors concluded that there was no increased death risk for patients who received 10–39 U PRBCs in the first 24 h compared to patients who received 0–9 U. This suggests that 40 U may be a threshold at which mortality increases significantly.
Morris et al. (2020) [98]	Retrospective Trauma Quality Improvement Program (TQIP) database analysis of 16,395 patients receiving MT defined as ≥4 U PRBCs in 4 h. This study looked at transfusion requirements in the first 4 h combined with decade of life to predict futility.	Mortality increased with age and transfusion requirement, which together may be used to guide prognosis. However, many older adults were resuscitated successfully, and thus age alone should not contraindicate large volume transfusion. However, as age increases, the number of units needed to approach futility decreases, and there is futility of transfusion past 51–60 U of PRBC within the first 4 h of admission in the octogenarian population. Also, giving more than 80 U PRBC in 4 h is associated with 100% mortality for all age groups.
Quintana et al. (2022) [20]	The TQIP database was used to find adult patients who received one or more U PRBC within the first 4 h of arrival from 2013 to 2017. Patients were analyzed based on the total amount of blood they received at 4 h and 24 h and whether they received blood in a 1:1 to 2:1 ratio of PRBC–plasma.	Mortality rate plateaued in transfusion volumes > 40.5 U at 4 h after admission and plateaued after 52.8 U at 24 h. For patients who received transfusion in a 1:1 to 2:1 ratio of PRBC–plasma, mortality rate plateaued after 39 U at 4 h and 53 U at 24 h.
Dorken Gallastegi et al. (2022) [25]	Examined data from the TQIP database between the years of 2013 and 2018. Patients receiving ultramassive (UMT), defined as ≥20 U PRBC in 24 h, were included. Transfusion volume was examined at 4 h and 24 h or time of death.	A transfusion rate of 7 U/hour for the first 24 h after arrival to the hospital is associated with 100% mortality. The authors conclude that defining futility should relate more to transfusion intensity than transfusion volume.
Anand et al. (2022) [16]	Retrospective TQIP database analysis of geriatric patients (≥65 years old). Patients were separated into 10-year-wide age groups. Futile resuscitation (FR) was defined as conditions leading to 90% mortality.	The authors conclude that transfusions > 40 U PRBC were futile for patients older than 65 years. The authors have described combined markers to define FR in geriatric patients. PRBC volumes at 4 h associated with FR were >30 U for 65–75-year-olds, >27 U for 75–85-year-olds, and >21 U for those older than 85 years. Also, increasing age was associated with increasing mortality among those who received emergency laparotomy or vasopressors, but did not reach FR.
Loudon et al. (2023) [12]	Retrospective observational study of 207 trauma patients who received transfusion in the first 4 h of care. Transfusion groups were defined as 2–9 U PRBC, 10–19 U PRBC (MT), >19 U PRBC (UMT) in 4 h.	Beyond 16 U PRBCs in the first 4 h, odds of mortality exceed survival. Survival approaches near zero with >36 U PRBC in the first 4 h. The authors termed efforts “heroic” at 16 U PRBC/4 h and “futile” at 36 U PRBC/4 h. Also, there was no survival beyond 67 U PRBC/4 h.
Ang et al. (2023) [24]	Retrospective cohort study of 1605 patients from 47 Level I or Level II trauma centers within one healthcare system. Data was taken from 2017 to 2019. Patients were stratified by age (16–30, 31–55, and ≥56) in an examination of percent mortality corresponding to varying transfusion volumes (≤ 24 units, 25–36 units, 37–48 units, 49–60 units, 61–72 units, 73–84 units, and >84 units where each unit was composed of an approximate 1:1:1 ratio of PRBCs, FFP, and PLTs).	There was a positive correlation between volume of transfusion and mortality and between age and mortality and a negative association between age and transfusion thresholds. The authors identify transfusion volumes for the age groups that indicate when the odds of mortality significantly increase with greater blood volumes transfused. These thresholds were 60 units for those aged 16–30 (30.3% mortality below the threshold and 67.7% mortality above the threshold), 48 units for those aged 31–55 (32.8% mortality below the threshold and 72.6% mortality above the threshold), and 24 units for those aged 56–100 (36.2% mortality below the threshold and 77.0% mortality above the threshold).
Clements et al. (2023) [11]	Retrospective observational study of 2299 trauma patients (median ISS 25, blunt injury 69%, 30-day mortality rate 22%) who received any blood products in the emergency department. First analysis compared those who received >50 U of blood components and those who received ≤ 50 U in the first 4 h. The second analysis compared those who received any WB during their resuscitation to those who received only blood components. One unit WB was defined as equivalent to 2.17 U blood components (1 U PRBC + 1 U FFP + 0.17 U PLT).	Survival rates in patients receiving >50 U of blood products in the first 4 h of care are as high as 50–60%, with survival still at 15–25% after 100 U. The authors concluded that futility should not be declared based on high transfusion volumes alone. Patients who received any WB (*n* = 1291) trended towards increased survival, but this was not statistically significant compared to the group who only received components. Therefore, futility could not be defined by transfusion volume alone.
Muldowney et al. (2023) [19]	Retrospective cohort study of 159 trauma patients (mean ISS 40, blunt trauma 34%, mortality rate 65%) who underwent UMTs defined as receiving ≥20 U PRBC and/or WB in the first 24 h.	50% of patients who received UMT received ≤ 30 U PRBC and WB. These UMT patients also had 65% mortality, which did not increase as more units of blood were given. The authors conclude that there is no transfusion cut-point for futility because the patients who received the most blood products still survived.
Schneider et al. (2023) [21]	Analysis of National Trauma Database to find all patients aged ≥18 who received ≥1 U PRBCs from 2017 to 2019; 61,676 patients were analyzed to arrive at 50% predicted mortality.	A mortality rate of 50% was predicted for all patients who received 31 U PRBCs. However, it was noted that for patients aged 80+, the 50% mortality rate was at 6 U PRBCs.
Major et al. (2025) [18]	Retrospective study of 3248 trauma patients aged ≥18 who underwent an operation and received ≥1 U blood products in 24 h. Data was taken from 7 US Level I trauma centers from 2016 to 2022. Patients were grouped into those who received UMT (≥20 U PRBCs in 24 h) and those who did not receive UMT.	The mortality rate for all included patients was 18.9%. Those who received UMT had a higher predicted mortality rate of 39.9% compared to the 6.8% mortality rate of those who did not receive UMT. In further stratification, the authors discovered that groups receiving 20–29 U PRBCs and 30–44 U PRBCs had similar risks of death. However, those who required ≥45 U PRBCs were correlated with a higher risk of death.
Van Gent et al. (2025) [22]	Prospective observational study conducted at seven trauma centers. Patients of interest were in need of hemorrhage control and blood transfusion. 1047 patients were included, and transfusion volumes were analyzed at 4 h.	Volumes < 110 U transfusion were not presented as futile and transfusion volumes of >110 U were associated with 100% mortality. Further analysis revealed that only preexisting risk factors could be used to predict futility.
Wallace et al. (2025) [23]	Analysis of TQIP database of patients aged ≥15 who received any volume of any blood product within 4 h from 2020 to 2022. 144,379 cases were reviewed to search for predictors of 24 h mortality based on quantities of transfusion.	The authors were unable to discern a difference in mortality predictability from either the quantities of PRBCs and low titer group O WB units transfused or the aggregate volume of transfused products. Thus, transfusion volumes could not effectively predict futility due to significant survival even at large volumes of blood transfused. 90% mortality at 24 h was achieved at >36 L transfused products or >56 U PRBC and WB.
